# NEK2 promotes the migration and proliferation of ESCC via stabilization of YAP1 by phosphorylation at Thr-143

**DOI:** 10.1186/s12964-022-00898-0

**Published:** 2022-06-15

**Authors:** Wei Su, Hao Hu, Qiurong Ding, Min Wang, Yan Zhu, Zhaochao Zhang, Zihan Geng, Shengli Lin, Pinghong Zhou

**Affiliations:** 1 Zhongshan Hospital Fudan University Endoscopy Center, Shanghai, 200032 China; 2 Shanghai Collaborative Innovation Center of Endoscopy, Shanghai, 200032 China; 3 Institute of Nutrition Science, Shanghai Institutes for Biological Sciences, Chinese Academy of Sciences, Shanghai, 200031 China; 4 Department of Gastroenterology, Hepatology and Nutrition, Shanghai Children’s Hospital, Shanghai Jiaotong University, Shanghai, 200062 China

**Keywords:** NEK2, Esophageal squamous cell carcinoma, YAP1, Epithelial-mesenchymal transition, Phosphorylation

## Abstract

**Background:**

Esophageal Squamous Cell Carcinoma (ESCC) was characterized as a regional-prevalent and aggressive tumor with high morbidity and mortality. NIMA-related kinase 2 (NEK2) is an interesting oncogene, the alteration of which leads to patients-beneficial outcomes. We aimed to explore the role of NEK2 in ESCC and excavate its mechanism.

**Methods:**

RNA-seq data were downloaded from TCGA and GEO and analyzed by R software. The protein levels were detected by immunohistochemistry (IHC) or western blot (WB), and mRNA expression was detected by qRT-PCR. The in vitro role of proliferation and migration was detected by Transwell migration assay and by colony formation assay, respectively. The in vivo roles were explored using a subcutaneous xenograft tumor model, where immunofluorescence (IF) and IHC were employed to investigate expression and localization. The interaction between proteins was detected by immunoprecipitation. The stability of proteins was measured by WB in the presence of cycloheximide.

**Results:**

A higher level of NEK2 was found in ESCC than normal esophageal epithelia in GEO, TCGA, and tissue microarray, which was associated with worse prognoses. The NEK2 knockdown impaired the proliferation and migration of ESCC, which also downregulated YAP1 and EMT markers like N-cadherin and Vimentin in vitro. On the contrary, NEK2 overexpression enhanced the migration of ESCC and elevated the levels of YAP1, N-cadherin, and Vimentin. Additionally, the overexpression of YAP1 in NEK2 knocked down ESCCs partly rescued the corresponding decrease in migration. The knockdown of NEK2 played an anti-tumor role in vivo and was accompanied by a lower level and nucleus shuffling of YAP1. In mechanism, NEK2 interacted with YAP1 and increased the stability of both endogenous and exogenous YAP1 by preventing ubiquitination. Moreover, the computer-predicted phosphorylation site of YAP1, Thr-143, reduced the ubiquitination of HA-YAP1, strengthened its stability, and thus influenced the migration in vitro.

**Conclusions:**

NEK2 is a prognostic oncogene highly expressed in ESCC and promotes the progression of ESCC in vitro and in vivo. Mechanistically, NEK2-mediated phosphorylation of YAP1 at Thr-143 protects it from proteasome degradation and might serve as a promising therapeutic target in ESCC.

**Video Abstract**

**Supplementary Information:**

The online version contains supplementary material available at 10.1186/s12964-022-00898-0.

## Background

Esophageal cancer ranked seventh most common cancer in 2020 and contributed one in eighteen pan-cancer deaths worldwide [1]. Esophageal Squamous Cell Carcinoma (ESCC) is the predominant histological subtype of esophageal cancer, which generally declines in incidence and remains high in mortality. Therefore, it is necessary to investigate those potent genes to benefit the overall survival of ESCC.

NEK2 is a long-discovered mitosis-associated oncogene promoting proliferation, epithelial-mesenchymal transition (EMT), drug resistance, oncogenesis, and immunity [2–4]. Besides, NEK2-mediated EMT also participated in migration, invasion, and cancer stemness in multiple cancers [5–7]. Recent reports indicated it phosphorated and stabilized specific substrates, such as PDL1 and Beclin-1, to evade immunity and autophagy-mediated drug resistance, respectively [8, 9]. Targeting NEK2 at its mechanism might benefit patients in chemoradiotherapy, targeted therapy, and immunotherapy in various cancers, including ESCC [10, 11].

Yes1-associated transcriptional regulator (YAP1) was identified as an essential growth initiator or enhancers in several solid tumors, such as hepatocarcinoma, gastric cancer, and colorectal cancer [12]. YAP1 mediated progression, tumorigenesis, stemness, and chemoresistance in ESCC [13–16]. Post-transcription modifications (PTMs) of YAP1 at different sites, the most recognized of which is phosphorylation, play diverse roles in the turnover and spatial regulation of YAP1 [17]. The functions of YAP1 and kinase NEK2 are significantly overlapped, but their association has not been elucidated.

Here, we investigated the expression and role of NEK2 in ESCC and its impact on YAP1 in vitro and in vivo. NEK2 promoted the proliferation, migration, and EMT by stabilization of YAP1. Furthermore, we also carried out a rescue assay to confirm NEK2 as a regulator of YAP1. In detail, NEK2 interacted with and prevented ubiquitin-mediated degradation of YAP1 by phosphorylating it at Thr-143.

## Methods

### Cell culture and antibody

The Eca-109 and immortalized esophageal mucosal cells (Het-1A) were gifted from Shanghai Medical College and Fudan University Shanghai Cancer Center, cultured in DMEM with 10% fetal bovine (FBS). KYSE-510 was purchased from Fuheng Biology and maintained in RPMI-1640 with 10% FBS. The source and application of antibody were: anti-NEK2 (sc-55601, Santa Cruz, 1:1000; abs136808, Absin, 1:1000), anti-YAP1 (sc-101199, Santa Cruz, 1:1000; GB111542, Servicebio, 1:1000; AF1615, Beyotime,1:1000), anti-GAPDH (AF0006, Beyotime, 1:1000) and anti-N-cadherin (GB111273, Servicebio, 1:1000), anti-Vimentin (GB11192, Servicebio, 1:1000), anti-Ubiquitin (AF0306, Beyotime, 1:1000), and anti-HA (AH158, Beyotime, 1:1000).

### Agents, kits, and plasmid

The agents, kits, and plasmids were listed as below: MG 132(HY-13259, MCE), cycloheximide (HY-12320, MCE), BeyoPure™ LB Broth (ST156, Beyotime), Fine Pure Endotoxin-free Extraction Kit (D907, GENFINE), puromycin (HY-B1743A, MCE), Pierce™ EZ-press RNA Purification Kit (B0004D, EZ Bioscience), SweScript RT I First Strand cDNA Synthesis Kit (G3331-100, Servicebio), 2 × SYBR Green qPCR Master Mix (G3321-05, Servicebio), Immunoprecipitation Kit (26149, Thermo Scientific™), lipofectamine 2000 (11668027, Thermo Scientific™), human NEK2 shRNA (Genechem), human YAP1^WT^ and mutated HA-YAP1 plasmid (Genomeditech), human siYAP1 (Genomeditech), and tissue microarray (HEsoS180Su11, Shanghai Outdo Biotech).

### Immunohistochemistry staining

The procedure contained antigen repairment, blockage by serum, combination with NEK2 (Absin, 1:300) or YAP1 (Servicebio, 1:1000) and secondary antibody, addition with DAB, redyeing, dehydration, separation. Each scoring equaled the intensity score multiplying its occupied area, where 1, 2, 3 were marked for the former and 1, 2, 3, 4 for the expansion of quartered proportion.

### Quantitative polymerase chain reaction

The total mRNA was extracted, quantified, reverse-transcripted into cDNA, and subscribed to qRT-PCR adhering to manufacturer instructions. The primers for NEK2 were F: 5’-TCCCCACTGAAATGAACTTTCT-3’, R:5’-CAGCTTGCTAAAGGAACGGA-3’, and those for GAPDH were F: 5’-CGAGATCCCTCCAAAATCAA-3’, R: 5’-TGTGGTCATGAGTCCTTCCA-3’.

### RNAi interference and stably knocked-down cell establishment

Three strings of shRNA targeting NEK2 (shRNA-1 CGTTCGTTACTATGATCGGAT, shRNA-2 GCAGACGAGCAAAGAAGAAAT, shRNA-3 CCTGTATTGAGTGAGCTGAA) targeting NEK2, together with shCon (TTCTCCGAACGTGTCACGT), were built into plasmids for transfection. The sequences of siRNA targeted YAP1 were #1 GAAGUAGUUUAGUGUUCUAtt, #2 GUUUCCCUGCUUUCCAGUUAAtt, and #3 GGAAGAGAUGAUGUAACUAtt. Knockdown validation was performed after 72 h post-transfection. The sequences of shRNA-3 and NEK2 were a reference for lentivirus construction. Puromycin was applied to the culture system post-infection to induce and maintain the expression.

### Clonal formation assay

500–1000 pretreated ESCCs were seeded and proliferated for 14 days. Then cells were fixed with 4% paraformaldehyde (PFA), dyed with crystal purple, and counted.

### Transwell migration assay

Approximately 10^6^ ESCC per chamber were seeded onto the upper membrane of Transwell and incubated in a serum-free medium to permit their migration into the lower one with 10% FBS for the indicated time. The migrated cells were fixed, dyed, and counted as above.

### Subcutaneous xenograft tumor model

Lentivirus-infected Eca-109 were injected into submucosae of nude mice. The dimensions of tumors were noted periodically, where volume was calculated as 0.5 × length × width^2^. The radiant efficiencies of bioluminescence were measured using IVIS^@^ Lumina K series III.

### Western blotting

The lysis of cells was quantitated and denaturalized for sampling. The samples were subscribed to SDS-PAGE and transmembrane for the indicated time. Then the membrane was blocked for non-specific antigens and incubated with primary and secondary antibodies. At last, the signals were indicated by chemiluminescence and quantified by intensity.

### Immunofluorescence

Paraffin-embed slides underwent dewaxing, antigen retrieval, non-specific antigen blockage, antibody incubation (NEK2, Absin, 1:100; HRP-labeling IgG; FITC), microwave processing, antigen blockage, another antibody incubation (YAP1, Beyotime,1:100; CY3-labeling IgG), nucleus dyeing, and finally observed with indicated excitation.

### Immunoprecipitation

Cell lysis was incubated with antibody (NEK2, Santa Cruz, 1:50; YAP1, Santa Cruz, 1:50; HA, Beyotime, 1:30) overnight at 4 ℃ and then linked to A/G beads for 2 h at room temperature. The mixture was washed and resuspended with NP-40 four times and denaturalized for immunoblotting.

### Statistics and analysis

Two groups were compared using Student’s t-test or by Mann–Whitney U test according to the homogeneity of variance. The differences in distribution were analyzed with the Chi-square test. ANOVA was used for comparing differences among multiple groups. We leveraged Cox regression and Kaplan–Meier plot to describe the prognoses and their hazards. The RNA-seq data were from authorized websites and interpreted by R software. GraphPad prism V8.0 was an alternative to revealing data.

## Results

### An elevated level of NEK2 was found in ESCC and associated with worse prognoses

**Fig. 1:**
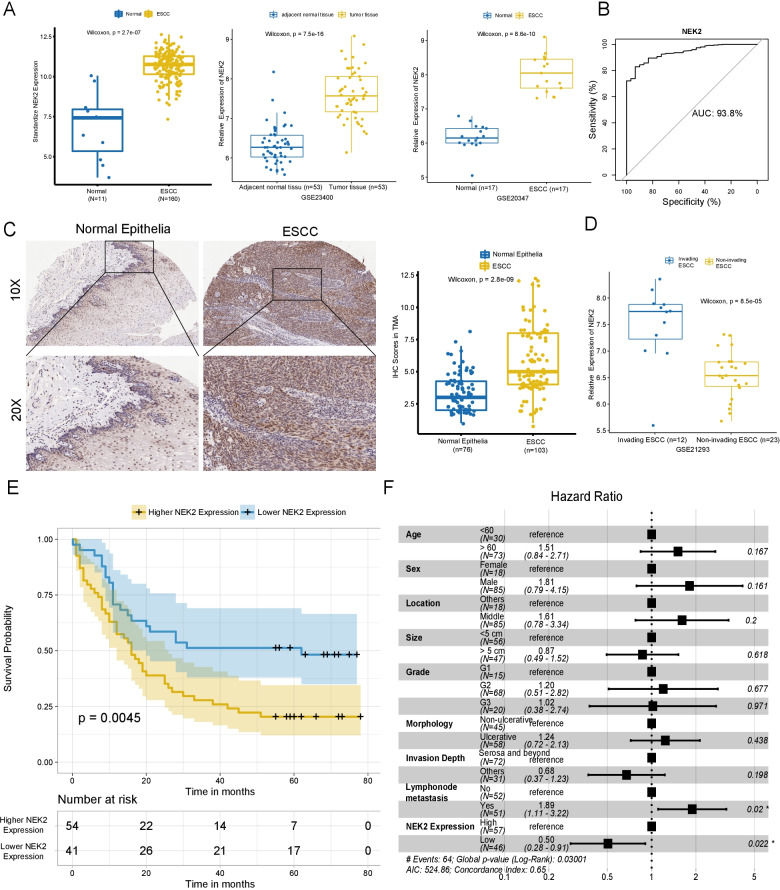
NEK2 was highly expressed in ESCC and indicated worse overall survival in patients. **A** The mRNA expression of NEK2 in ESCC tissues was varied from 2.475 to 5.205 folds of those in normal esophageal epithelia in TCGA-ESCA, GSE23400, GSE20347. **B** NEK2 presented as a reliable cancerous biomarker with distinguished AUC at 93.8% in the TCGA cohort. **C** NEK2 was expressed abundantly throughout the ESCC and its level was higher than normal epithelia in tissue revealed by TMA, most of which were confined to the basal layer in the latter (*P* < 0.001, Mann–Whitney U test). **D** Higher expression of NEK2 was more frequently found in invading ESCC than non-invading ones in GSE21293(*P* < 0.001, Wilcoxon test). **E** Higher expression of NEK2 led to worse overall survival in ESCC patients. **F** Higher expression of NEK2, as well as lymph node invasion, were two independent predictors of ESCC mortality. *TCGA* The Cancer Genome Atlas; *ESCA* esophageal carcinoma; *AUC* area under the curve; *TMA* tissue microarray

We compared the differences in mRNA levels between ESCC and normal esophageal epithelia, TCGA led the widest gap with 5.205 folds higher in ESCC, followed by GSE20347 and GSE23400 at 3.630 and 2.457 (all *P* < 0.0001, Fig. 1A), respectively. In addition, NEK2 expression also showed good resolving power with a ROC of 93.8% (Fig. 1B).

Then we investigated the protein expression in TMA. The median IHC score was 5 (IQR: 4–8), significantly higher than those 3 (IQR: 2–4.25) in para-tumor epithelia (*P* < 0.001), where the NEK2 was most expressed around the base layer (Fig. 1C). Moreover, the mRNA levels were higher in invading ESCC than those in non-invasive ones in GSE21293 (*P* < 0.001, Fig. 1D). Consistently, an elevated level of NEK2 was positively associated with undifferentiation, lymph node invasion, and advance in the 8th AJCC stage (*P* = 0.001, *P* = 0.029, and *P* < 0.001, respectively), revealing the association with lymphatic invasion and worse prognosis (Table 1). Furthermore, increased NEK2 led a 5-year survival of 20.3% (95%CI 12%–34.5%), while that increased to 48.2% (95%CI 35%–66.5%) in patients with decreased NEK2 expression in Fig. 1E (*P* = 0.0045). Additionally, an increased level of NEK2 was screened out as an independent factor of mortality in Fig. 1F, determining it as an indicator of poor prognoses.

**Table1:** Correlation between NEK2 expression and clinicopathological parameters

	Higher expression of NEK2 (n = 57)	Lower expression of NEK2 (n = 46)	*P*
Sex, Male (%)	49 (86.0)	36 (78.3)	0.446
Age (mean (SD))	66.61 (9.47)	64.61 (7.40)	0.243
Size (mean (SD))	4.70 (1.79)	4.83 (1.77)	0.717
Location (%)			0.779
Middle	46 (80.7)	39 (84.8)	
Others	10 (19.3)	8 (15.2)	
Morphology (%)			0.111
Plaque	11 (19.3)	5 (10.9)	
Fungoid	12 (21.1)	14 (30.4)	
Ulcerative	0 (0.0)	3 (6.5)	
Medullary	34 (59.6)	24 (52.2)	
Grade (%)			0.001
G1	2 (3.5)	13 (28.3)	
G2	40 (70.2)	28 (60.9)	
G3	15 (26.3)	5 (10.8)	
Invasion depth (%)			0.557
Submucosal	1 (1.8)	2 (4.3)	
Muscular	14 (24.6)	14 (30.4)	
Serosa	42 (73.7)	30 (65.2)	
Lymph node metastasis			0.029
Yes	34 (59.6)	17 (37.0)	
No	23 (40.4)	29 (63.0)	
T stage (%)			0.462
T1	1 (1.8)	2 (4.3)	
T2	13 (22.8)	13 (28.3)	
T3	41 (71.9)	31 (67.4)	
T4	2 (3.5)	0 (0.0)	
N stage (%)			0.065
N0	23 (40.4)	29 (63.0)	
N1	20 (35.1)	11 (23.9)	
N2	10 (17.5)	6 (13.0)	
N3	4 (7.0)	0 (0.0)	
Metastasis	0	0	
AJCC* stage (%)			0.001
I	1 (1.8)	5 (10.9)	
II	22 (38.6)	24 (52.2)	
III	27 (47.4)	17 (37.0)	
IV	7 (12.3)	0 (0.0)	

^*^AJCC The 8th Version of American Joint Committee on Cancer

### NEK2 influenced the migration and proliferation of ESCC in vitro

**Fig. 2:**
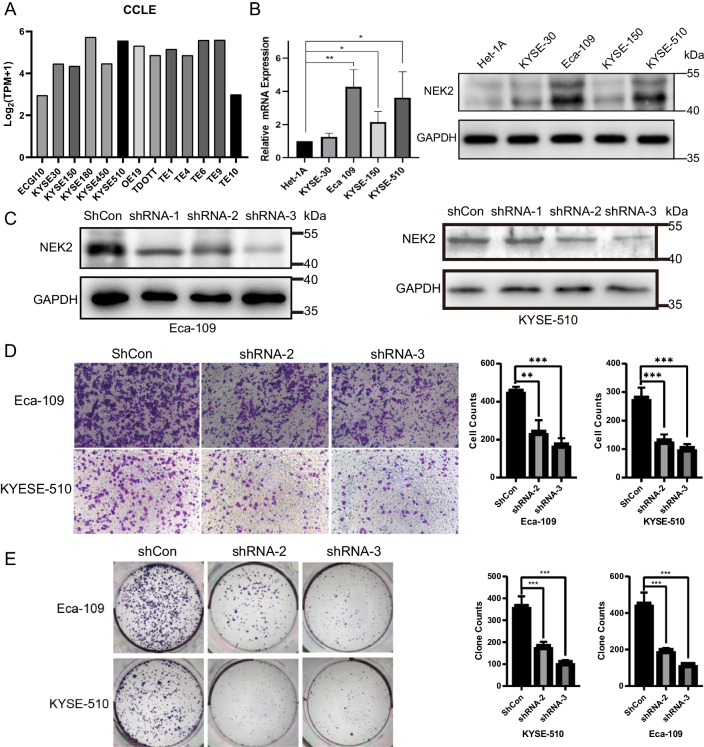
Knockdown of NEK2 leads to the impairment of migration and proliferation of ESCC in vitro. **A** The RNA-seq data of CCLE showed various expressions of NEK2 in ESCC. **B** The expression of NEK2 in ESCC was significantly higher than immortalized esophageal epithelia Het-1A in transcriptional and translational levels. **C** Both shRNA-2 and shRNA-3 in KYSE-510, as well as all strings in Eca-109, yielded efficacy in knocking down NEK2 at the translational level. **D** Knockdown of NEK2 in both cells showed declined migration in Transwell assay. **E** Knockdown of NEK2 in both cells exhibits decreased proliferation in clone formation assay. All experiments were carried out independently for three times. *P < 0.05, **P < 0.01, ***P < 0.001. *CCLE* Cancer Cell Line Encyclopedia

NEK2 expression was relatively high but uneven in ESCC cell lines in CCLE (Fig. 2A). Comparing to Het-1A, the expression of NEK2 in Eca-109 and KYSE-510 were detected higher in transcriptional (4.27 ± 1.04 folds, *P* = 0.006 and 3.62 ± 1.56 folds, *P* = 0.04) and translational level (2.43 ± 0.3 folds, *P* < 0.001 and 2.29 ± 0.17 folds, *P* = 0.001), thus selected for further research (Fig. 2B). Figure 2C validated interfering with NEK2 of shRNA-2 and shRNA-3 in Eca-109 (1 VS 0.55 ± 0.06 and 1 VS 0.26 ± 0.05, all *P* < 0.001) and KYSE-510 (1 VS 0.50 ± 0.07 and 1 VS 0.32 ± 0.05, all *P* < 0.001). The knockdown groups in Eca-109 showed fewer transmembrane cells (240.3 ± 62.2 and 173 ± 34.7) than those of control (456.3 ± 21.6, *P* = 0.015 and *P* < 0.001), and the circumstance were alike in KYSE-510 (129.7 ± 22 and 102.3 ± 15.5 VS 272 ± 36.1, all *P* < 0.001, Fig. 2D). Moreover, knocking down NEK2 deaccelerated the colony formation from 450.3 ± 62 to 196.3 ± 12.5 and 119 ± 5.3 in Eca-109 (all *P* < 0.001) and in KYSE-510, they descended from 364 ± 45.2 to 182 ± 20.1 and 108.3 ± 10.8 (all *P* < 0.001, Fig. 2E).

### NEK2 regulated YAP1 and impacted EMT of ESCC in vitro

**Fig. 3:**
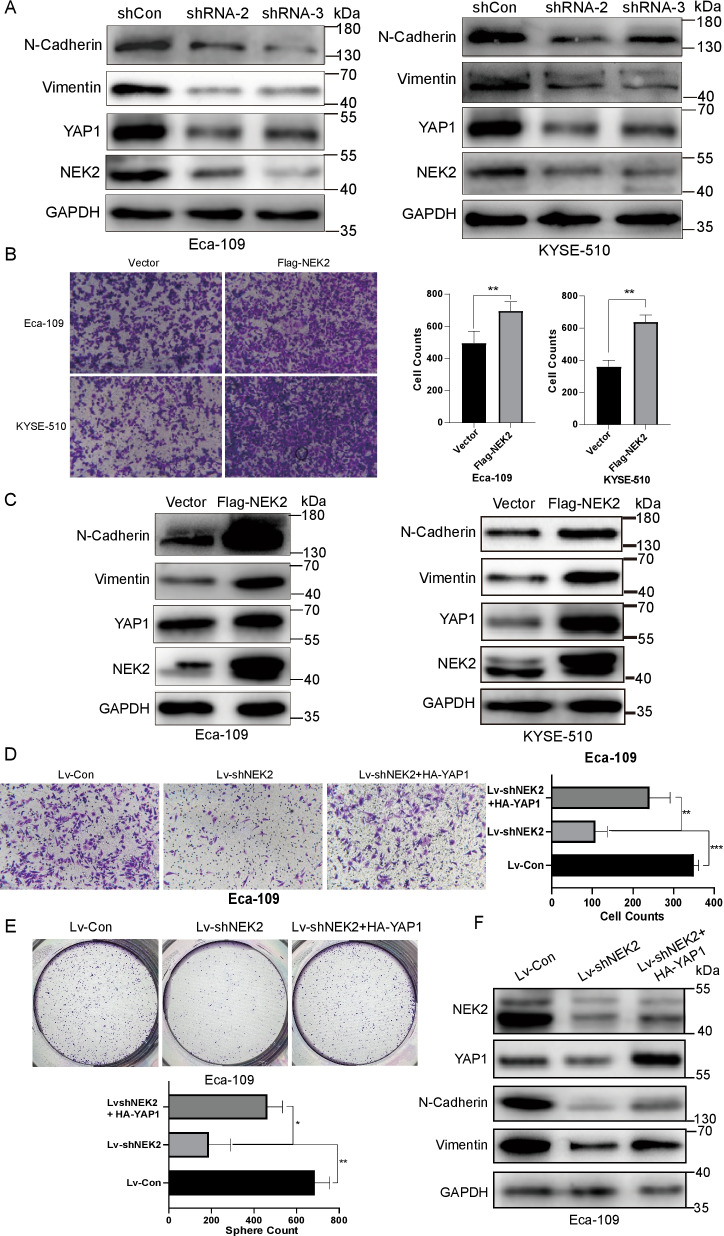
NEK2 regulated expression of YAP1 and affected EMT of ESCC in vitro. **A** Silence NEK2 with two shRNA both decreased the expression of YAP1 and EMT biomarkers like N-cadherin and Vimentin. **B** Overexpression of NEK2 enhanced the migration in ESCC by Transwell assay. **C** Overexpression of NEK2 increased the expression of YAP1, N-Cadherin, and Vimentin. **D** The impairment of migration caused by Lv-shNEK2 could be partly recovered by the introduction of HA-YAP1 in Eca-109. **E** The reduction of N-Cadherin and Vimentin due to knockdown of NEK2 could be incompletely rescued with the elevation of YAP1 in Eca-109. All images represented one of three independently repeated experiments. **P < 0.01, ***P < 0.001

Besides the impacts above, silencing NEK2 on both ESCCs deregulated YAP1 and EMT biomarkers, Vimentin, and E-cadherin (Fig. 3A). On the opposite, NEK2 overexpression promoted the migration in Eca-109 (695 ± 56.9 VS 495 ± 73.4, *P* = 0.0204), and enhanced migration was also seen in KYSE-510 (360 ± 39.4 VS 638.3 ± 43.6, *P* = 0.001, Fig. 3B). Meanwhile, we also found elevated levels of YAP1, N-Cadherin, and Vimentin in both ESCCs (Fig. 3C). For the convenience of further study, shRNA-3-based lentiviruses infected Eca-109 to establish a stably NEK2 knockdown model as Lv-shNEK2 and Lv-Con were built as its control. As shown in Fig. 3D, the formation clone in the Lv-shNEK2 group were108 ± 28.8, significantly fewer than Lv-Con (358 ± 11.9, *P* < 0.001). Introducing HA-YAP1 plasmid to the Lv-shNEK2 group partly rescued the depressed migration at 241 ± 50.4 (*P* = 0.008) but it was still lower than Lv-Con (*P* = 0.01). Likewise, the expression of exogenous YAP1 partly elevated repressed proliferation in Lv-shNEK2 group (463.67 ± 69.9, *P* = 0.015), while still lower than Lv-Con (686 ± 70.2, *P* = 0.037). Correspondingly, N-cadherin and Vimentin, which were downregulated by NEK2, were partly restored by overexpression of YAP1 (Fig. 3F).

### NEK2 influenced the growth of ESCC and co-existed with YAP1 in vivo

**Fig. 4:**
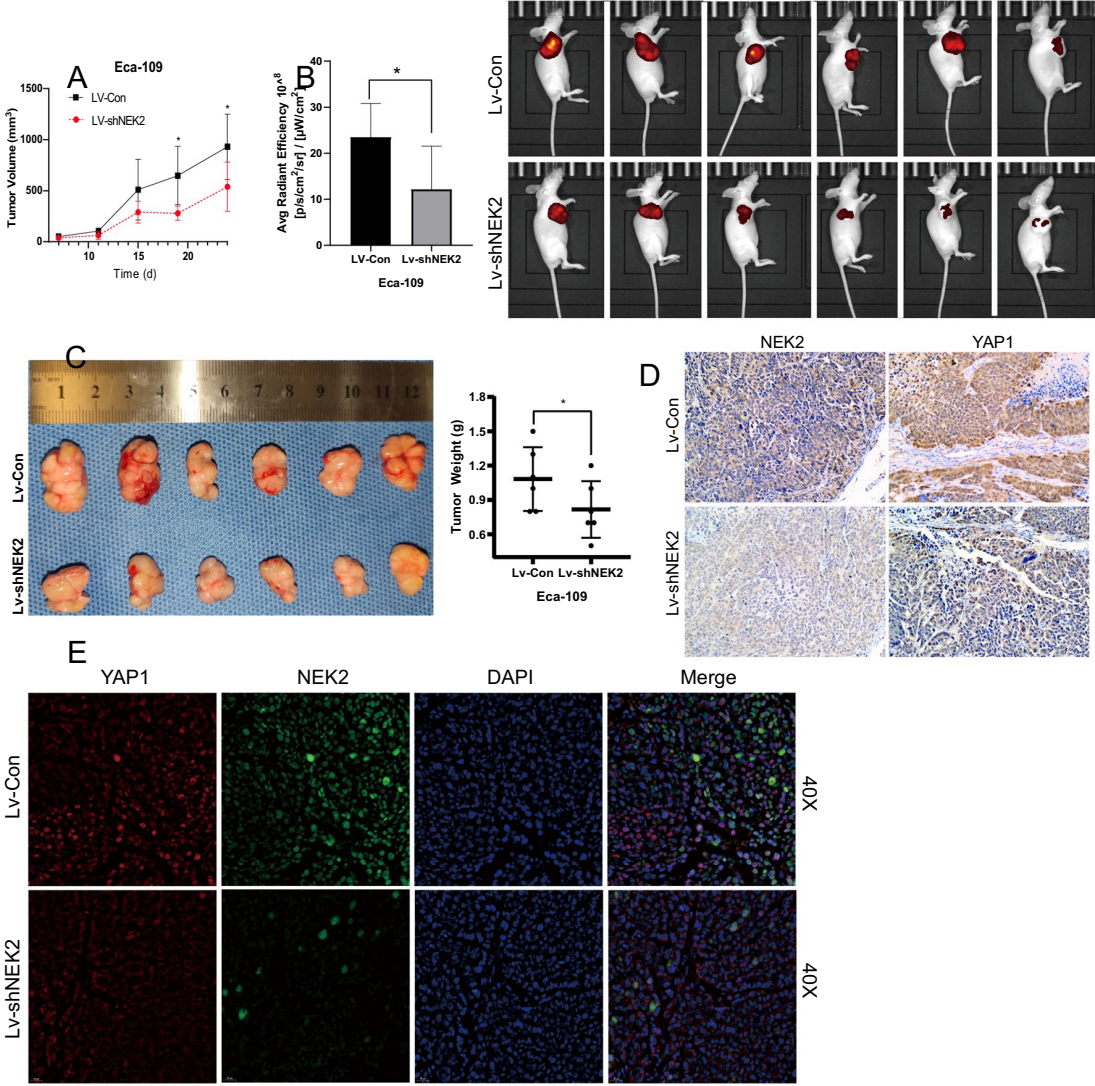
NEK2 influenced the oncological effect of ESCC and co-existed with YAP1 in vivo. **A** Knockdown of NEK2 decelerated the growth rate of Eca-109 in vivo. **B** Lv-NEK2 group had a compromised manifestation in average radiant efficiency and size than those in the Lv-Con group in vivo imaging. **C** Knockdown of NEK2 reduced the tumorigenesis of Eca-109 presented in size and weight. **D** The decreased NEK2 was accompanied by reduced expression and nucleus shuffling of YAP1 by IHC staining. **E** The downregulated expression of NEK2 simultaneously manifested with less expression and nucleus shuffling of YAP1 by IF imaging. *P < 0.05; *IHC* immunohistochemistry; *IF* immunofluorescence

The xenografted tumor in Lv-shNEK2 exhibited a slower growth than Lv-Con (Fig. 4A). Figure 4B, C showed the radiant efficiency and weight of the Lv-shNEK2 group were both significantly compromised than Lv-Con (23.52 ± 7.33 VS 12.18 ± 9.34, *P* = 0.041;1.08 g VS 0.75 g, *P* = 0.0302). Then IHC revealed the predominant location of NEK2 in the cytoplasm, the decrease of which was also accompanied by synchronized declined levels of YAP1 and fewer locations in the nucleus (Fig. 4D). A similar relationship between NEK2 and YAP1 frequently appeared in the depiction of IF staining, which indicated the in vivo function of NEK2 might relate to YAP1 (Fig. 4E).

### NEK2 phosphorylated and stabilized YAP1 at Thr-143 in vitro

**Fig. 5:**
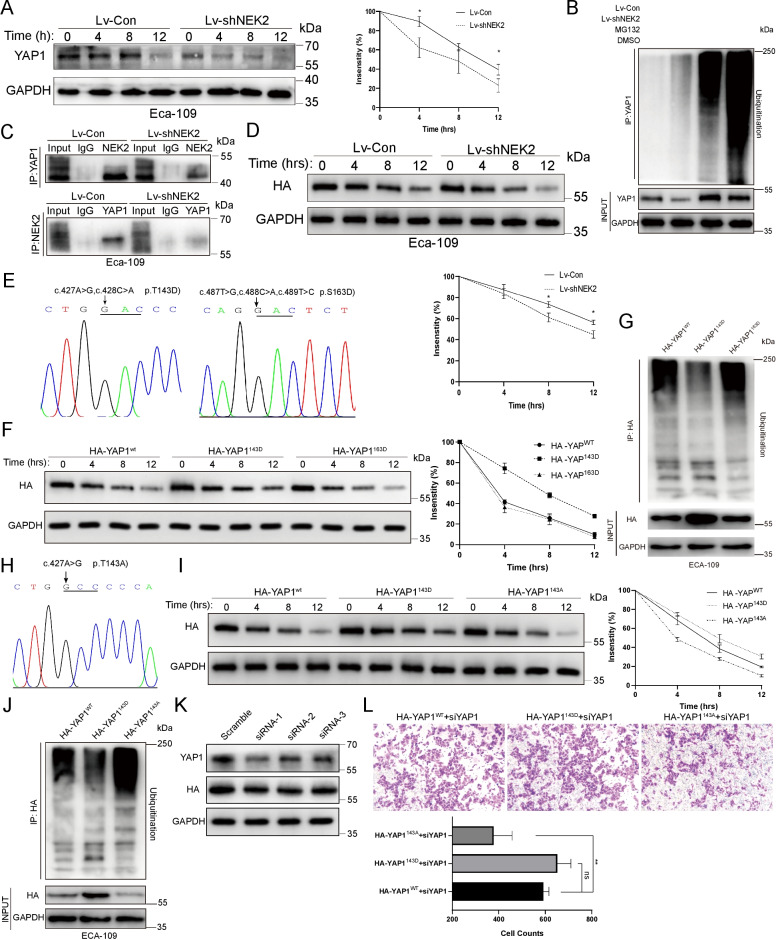
NEK2 stabilized YAP1 and hindered its ubiquitination by phosphorylating it at Thr-143. **A** Endogenous YAP1 was subscribed to faster degradation in 12 h treatment of cycloheximide (20 μg/mL) between the Lv-Con and Lv-shNEK2 group. **B** The YAP1 underwent stronger ubiquitination in the Lv-shNEK2 group, which was more apparent in the presence of pretreatment MG132 (10 μM, 4 h) but still more intensified than that of the Lv-Con group. **C** NEK2 was pulled down by YAP1 in IP and vice versa, which was weaker in the Lv-shNEK2 group in Eca-109 in vitro. **D** The stability of Exogenous HA-YAP1 was also declined after knocking down on YAP1. **E** Sequencing map of mutation of Thr-143 and Ser-163 into Asp-143 and Asp-163, respectively. **F** The stability of HA-YAP1 was significantly stronger in the HA-YAP1^143D^ group than HA-YAP1^WT^ group and HA-YAP1^163D^ group. **G** The HA-YAP1^143D^ was less ubiquitinated than HA-YAP1^WT^ and HA-YAP1^163D^ group with pretreatment of MG132 (10 μM, 4 h). **H** Sequencing map of mutation of Thr-143 into Ala-143. **I** The HA-YAP1 was subscribed to the fastest degradation when its Thr-143 was mutated into alanine, while the lowest rate was found with the mutation to aspartic acid. **J** The HA-YAP1^143A^ group presented the most ubiquitinated, with MG132 (10 μM, 4 h) as previous, followed by the HA-YAP1^WT^ group and HA-YAP1^143D^ group. **K** The 3’-UTR-oriented siRNA was proved efficient to interfere with endogenous YAP but not exogenous YAP1. **L** The Mutation of Thr-143 into alanine on exogenous YAP1 limited the immigration of ESCC in vitro in the presence of siRNA-1 for endogenous YAP1. All experiments were repeated independently for three times. *P < 0.05, **P < 0.01, *ns* not significant, *IP* immunoprecipitation

YAP1 protein underwent faster degradation in the Lv-shNEK2 group, confirming the stabilizing role of NEK2 on the YAP1 turnover (Fig. 5A). Meanwhile, the Lv-shNEK2 group was more ubiquitinated than the control while the gap was more obvious but could not be narrowed down in the presence of proteasome inhibitor MG-132, demonstrating the participation of ubiquitination (Fig. 5B). Based on reports NEK2 mediated the crosstalk of phosphorylation and ubiquitination on substrates [9, 18, 19], the combination of NEK2 and YAP1 should be proved to allow the occurrence of phosphorylation. It turned out that NEK2 was precipitated by YAP1-connect beads and vice versa, which became weaker in the Lv-shNEK2 group, confirming their physical contact in vitro (Fig. 5C).

Given that YAP1 was stabilized by NEK1 via phosphorylation [20], we hypothesized that phosphorylation by NEK2 attributes to the stabilization of YAP1. Then employing GPS 5.0 algorithm [21], we predicted the phosphorylation sites of YAP1 by NEK2 to design mutated fusion YAP1 to facilitate further researches. As a premise, the stability of exogenous HA-YAP1 was also decreased in Lv-shNEK2 (Fig. 5D). Thr-143 and Ser-163 within YAP1, which were the highest-ranked sites in GPS 5.0, were mutated to phospho-mimetic aspartic acid (Asp), namely HA-YAP1^143D^ and HA-YAP1^163D^, and built into plasmids to check their stability (Fig. 5E). The YAP1^143D^, instead of Ser-163, slowed the degradation of the exogenous YAP1 (Fig. 5F) and ranked the least ubiquitinated group compared with YAP1^WT^ and YAP1^163D^ (Fig. 5G), verifying the ubiquitin-free role of phosphorylation of Thr-143. On the contrary, the HA-YAP1^143A^, as a dephosphorylation mimetic (Fig. 5H), led to the fastest degradation and maximum ubiquitination (Fig. 5I, J), confirming the protective role of Thr-143 phosphorylation again. At last, we explored the biological function of exogenous mutated YAP1 with interfering endogenous one using siRNAs targeting 3’-UTR. The efficiency of siRNA-1 was relatively satisfying with 60% of total YAP1 without disruption of exogenous one (Fig. 5K). The transmembrane cell of the HA-YAP1^143A^ group declined significantly compared with the HA- YAP1^143D^ (378 ± 79.8 VS 651.7 ± 58.1, *P* = 0.003) and HA- YAP1^143D^ (378 ± 79.8 VS 591.7 ± 24.34, *P* = 0.01), confirming the positive role of phosphorylation at Thr-143 in migration once again (Fig. 5L).

## Discussion

ESCC claimed the most prevalence and deaths of esophageal cancer worldwide [22, 23]**.** PD-L1 positivity was seen in up to 43.7% of ESCC tissues and several PD-L1-targeting combined therapy attained impressive overall survival benefits [24–27]. NEK2 is a well-recognized oncogene and a potent enhancer of PD-L1 in pancreatic ductal adenocarcinoma and the alteration of NEK2 orchestrated other phenotypes in migration, and chemoresistance [8, 28, 29]. Similarly, depletion of NEK2 may also aid the PD-L1 treatment in ESCC in other ways.

In this study, the repressed phenotypes like migration, proliferation, and EMT were induced by downregulating NEK2, presenting a therapeutic value in ESCC. Additionally, NEK2 served as an independent prognostic factor and was scarcely expressed in esophageal epithelia but elevated in ESCC, leaving a treatment window for patients. It is not surprising because NEK2 was involved in malignant behavior in triple-negative breast cancer, lung cancer, hepatocellular cancer, and multiple myeloma, and indicated worse prognoses [30–33]. The CIN (chromosomal instability) caused by aberrant NEK2 expression may be the root, which drives genome-wide hypomethylation, development, and progression of ESCC [34]. Beyond phenotype, YAP1 was a downstream effector of NEK2, where the rescued expression of YAP1 recovered the proliferation and migration of ESCC in vitro*.* We also observed NEK2 knockdown paralleled decreasing YAP1 and its nucleus shuffling in vivo. However, it’s hard to differ whether the spatial regulation resulted from the accumulation of cytoplasmicYAP1. However, no research has unveiled on how NEK2 regulated YAP1 till now.

The mechanism was unfolded in three steps. Firstly, the relatively high level of YAP1 was owing to the stabilization and ubiquitin-free roles of NEK2. The pattern was similar to the previous report where NEK2 protects its substrates directly or indirectly from ubiquitination and proteolysis, just as Beclin-1, SRSF1, and β-catenin [9, 35, 36]. Secondly, we found a new interaction between YAP1 and NEK2. It’s hinted in previous reports of NEK1 and YAP1, and the combination was a premier for phosphorylation [20]. Thirdly, the PTM of YAP1 induced by NEK2 strengthened the stability of YAP1. The PTMs of YAP1 affect its expression and spatial regulation and were critical in the realization of oncological functions like migration, EMT, and chemoresistance [17, 37–39]. Classically recognized PTM of YAP1 is phosphorylation at Ser-127, which enhances nucleus shuttling and activates the pathway [40]. However, the sites and their effects on YAP1 are diverse. For instance, the formation of phosphodegron by Ser-381 phosphorylation recruited SCFβ^Trcp^ mediated ubiquitination while Y315 and Y357 phosphorylation may increase the stability of YAP1 [20, 40, 41]. Herein, phosphorylation at Thr143 stabilized the YAP1 by preventing its ubiquitination in ESCC. The phenomenon was supported by the reports where NEK2 mediated stabilization of its phosphorylated substrates, but contrary to ubiquitination-degradation induced by Ser-381 phosphorylation [9, 42, 43]. In a wider scope, the phosphorylation of certain proteins may unusually slow the turnover and stabilize its substrate against the odds [44]. We hypothesized that Ser-143, which was close to Ser-127, shared similar biological significance in spatial regulation. Whether the sites be ubiquitinated or not might be decided by the structure of the context.

Some limitations still existed. Firstly, the orthotopic xenograft model was not established because of high mortality in the process. Secondly, other downstream genes of YAP1 in ESCC were not detected, one of which was SOX-9 in a previous study [45]. Thirdly, there is a lack of direct evidence like in vitro phosphorylation experiments due to difficulties in developing the antibody against Thr-143.

Herein, NEK2 phosphorylated YAP1 at Thr-143 to increase its stability in ESCC. Given such phosphorylation promotes the progression of ESCC, the roles of other important PTMs in YAP1, like acetylation and glycosylation, should be explored in the development and progression of ESCC. The management of YAP1 by PTMs regulation might be a new strategy to combat ESCC.

## Conclusion

We revealed the prognostic significance of elevated expression of NEK2 and its roles in proliferation, migration, and EMT in ESCC. Mechanistically, we suggested NEK2 interacted with and phosphorylated YAP1 at Thr-143, which alleviated its ubiquitination in vitro, promoted stability, and thus mediated the progression of ESCC.

## Data Availability

The datasets used and/or analyzed during the current study are available from the corresponding author on reasonable request.

## Abbreviations

ESCC: Esophageal squamous cell carcinoma
NEK2: NIMA-related kinase 2
GEO: Gene expression omnibus
TCGA: The Cancer Genome Atlas
YAP1: Yes1-associated transcriptional regulator
EMT: Epithelial-mesenchymal transition
CCLE: Cancer Cell Line Encyclopedia
TMA: Tissue microarray
PCR: Quantitative polymerase chain reaction
IHC: Immunohistochemistry
WB: Western blotting
IF: Immunofluorescence
IP: Immunoprecipitation
ESCA: Esophageal carcinoma
ns: Not significant
AJCC: American Joint Committee on Cancer
